# Development of Masitinib Derivatives with Enhanced M^pro^ Ligand Efficiency and Reduced Cytotoxicity

**DOI:** 10.3390/molecules28186643

**Published:** 2023-09-15

**Authors:** Cintia A. Menendez, Adil Mohamed, Gustavo R. Perez-Lemus, Adam M. Weiss, Benjamin W. Rawe, Guancen Liu, Alex E. Crolais, Emma Kenna, Fabian Byléhn, Walter Alvarado, Dan Mendels, Stuart J. Rowan, Savaş Tay, Juan J. de Pablo

**Affiliations:** 1 Pritzker School of Molecular Engineering, University of Chicago, 5640 South Ellis Avenue, Chicago, IL 60637, USAgrp@uchicago.edu (G.R.P.-L.); brawe@uchicago.edu (B.W.R.); stuartrowan@uchicago.edu (S.J.R.); amannie@uchicago.edu (S.T.); 2 Department of Chemistry, University of Chicago, 5735 South Ellis Avenue, Chicago, IL 60637, USAguancen123@uchicago.edu (G.L.); acrolais@uchicago.edu (A.E.C.); 3 Argonne National Laboratory, 9700 South Cass Avenue, Lemont, IL 60439, USA

**Keywords:** masitinib derivatives, M^pro^ inhibitors, SARS-CoV-2

## Abstract

Recently, a high-throughput screen of 1900 clinically used drugs identified masitinib, an orally bioavailable tyrosine kinase inhibitor, as a potential treatment for COVID-19. Masitinib acts as a broad-spectrum inhibitor for human coronaviruses, including SARS-CoV-2 and several of its variants. In this work, we rely on atomistic molecular dynamics simulations with advanced sampling methods to develop a deeper understanding of masitinib’s mechanism of M^pro^ inhibition. To improve the inhibitory efficiency and to increase the ligand selectivity for the viral target, we determined the minimal portion of the molecule (fragment) that is responsible for most of the interactions that arise within the masitinib-M^pro^ complex. We found that masitinib forms highly stable and specific H-bond interactions with M^pro^ through its pyridine and aminothiazole rings. Importantly, the interaction with His^163^ is a key anchoring point of the inhibitor, and its perturbation leads to ligand unbinding within nanoseconds. Based on these observations, a small library of rationally designed masitinib derivatives (**M1**–**M5**) was proposed. Our results show increased inhibitory efficiency and highly reduced cytotoxicity for the **M3** and **M4** derivatives compared to masitinib.

## 1. Introduction

The severe acute respiratory syndrome coronavirus 2 (SARS-CoV-2) was identified in December 2019 as responsible for the viral pneumonia outbreak that commenced in Wuhan City, Hubei Province, China. This outbreak rapidly spread across the world, causing the ongoing COVID-19 pandemic [1,2,3,4]. Several vaccines are now available, but new and more infectious mutants capable of evading vaccines continue to pose serious concerns. The search for new treatment options for COVID-19 and other coronaviruses is critical, as is development of broad-spectrum antivirals.

Following fusion of the viral envelope with the host cell membrane, the viral +ssRNA genome is released into the host cell cytoplasm. It is then translated into around 30 proteins, 16 of which are initially part of two polyproteins (ORF1a and ORF1b) that must be cleaved into individual pieces to support the next steps of the infection. This cleavage is guided by two viral encoded proteases, the papain-like protease, PLpro (NSP3) [5], and the viral main protease, known as M^pro^, 3CLpro, or NSP5 [6].

From the complete viral proteome, only a handful of proteins have been selected as targets for inhibition compounds in experiments. For example, the viral RNA-dependent RNA polymerase (RdRp) is the original target of Remdesivir (GS-5734), the first approved antiviral agent in the U.S. against SARS-CoV-2. Remdesivir has been reported to shorten COVID-19 hospitalization times, but it failed a large clinical trial in hospitalized patients [7]. Lagevrio (Molnupiravir) is an antiviral that targets the same RdRp complex as Remdesivir, and phase 2/3 clinical trials show evidence of its efficacy against different strains of SARS-CoV-2 virus. It was recently authorized for emergency use in the United States. Paxlovid (nirmatrelvir; ritonavir) is an inhibitor of the viral Main protease (M^pro^) [8] and was the second approved antiviral agent against SARS-CoV-2 in the U.S. The phase 2/3 clinical trial has shown promising results, but there is limited information about its safety and effectiveness against COVID-19 and it is still being evaluated.

Conventional drug design and development strategies require lengthy synthesis and screening of lead compounds, pre-clinical and clinical testing of efficacy, and substantial funding. The clinical screening time can be reduced considerably by using already approved pharmaceutical compounds that can be re-purposed. Indeed, Remdesivir [7], Lagevrio, and Paxlovid [8] were all identified as COVID-19 anti-viral compounds via drug re-purposing studies. Sarkar, et al. also explored the potential of repurposed antiviral compounds (peptidomimetic and non-peptidic) against the SARS-CoV-2 main protease (Mpro) [9]. A systematic in silico drug repurposing screening of compounds from the DrugBank database identified 22 drugs or experimental compounds as promising SARS-CoV-2 main protease (Mpro) inhibitors and discussed their potential as polypharmacologic agents [10]. The repurposing of fullerenes C60 and C70 was also considered based on in silico studies [11].

Computer-aided drug design (CADD), an interdisciplinary approach that combines computational biology with medicinal chemistry, is an important complement to experimental studies in that it accelerates drug design through the rapid identification of new targets. CADD can also reveal the mechanisms of action of small molecules and identify structural modifications that could serve as targets for optimization [12,13,14]. More specifically, molecular simulations are important as a guide in improving the binding properties and efficacy of lead compounds through the use of detailed molecular models. One of the main advantages of molecular dynamics or Monte Carlo simulations compared to other approaches, such as molecular docking simulations, is the explicit treatment of structural flexibility and entropic effects [14].

Recently, a high throughput screen of 1900 clinically used drugs identified masitinib, an orally bioavailable tyrosine kinase inhibitor, for treatment of COVID-19. Masitinib acts as a potent broad-spectrum inhibitor for human coronaviruses, including SARS-CoV-2 and its variants of concern [6]. It was shown that masitinib acts as a competitive inhibitor for the SARS-CoV-2 main protease (M^pro^ or 3CLpro), and the complex crystallographic structure indicates that masitinib binds non-covalently between domains I and II of M^pro^ and blocks the key catalytic residues Cys145 and His^41^ [6].

In this work, we rely on molecular modelling with advanced sampling methods to develop a deeper mechanistic understanding of masitinib’s mechanism of M^pro^ inhibition. With the objective of developing new analogues of masitinib with increased inhibitory potency and selectivity for the viral target (M^pro^), it is of interest to determine the minimal portion of the molecule (fragment) that is responsible for most of the interactions developed within the masitinib-M^pro^ complex. The rationale behind this approach is based on two considerations: first, having completely understood masitinib’s mechanism of M^pro^ inhibition, it might be easier to construct a completely new library of derivatives with differential structural patterns comparable to kinase inhibitors. Second, the optimization process of DMPK (drug metabolism and pharmacokinetics) properties usually involves an increase in molecular weight. Thus, starting from smaller but more efficient fragments would be beneficial in subsequent steps. We find that masitinib forms highly stable and specific H-bond interactions with M^pro^ through its pyridine and aminothiazole rings. Of importance, the interaction with His^163^ is a key anchoring point of the inhibitor, and its perturbation leads to ligand unbinding within nanoseconds. To corroborate these observations in experiments, a small library of masitinib derivatives (**M1**–**M5**) was synthesized and tested in a HCoV-OC43 live-cell infectivity assay, as well as in in vitro SARS-CoV-2 protease (M^pro^ or 3CL^pro^) and cytotoxicity assays. Our results show increased inhibitory potency and highly reduced cytotoxicity for the **M3** compared to masitinib. Moreover, in terms of ligand efficiency, LE [15], an improvement of roughly 25% and 50% is observed for **M3** and **M4**, respectively, compared to masitinib. Considering the lower cytotoxicity exhibited for **M3** and **M4**, in addition to the fact that smaller but more efficient binders are often desirable as they have a greater successful rate advancing through the lead optimization stage, the **M3** and **M4** derivatives could be considered as good starting options for further lead optimization cycles. 

## 2. Results

### 2.1. Molecular Simulation of M^pro^-Masitinib Complex: Exploration of Ligand–Protein Key Interactions

**Figure 1:**
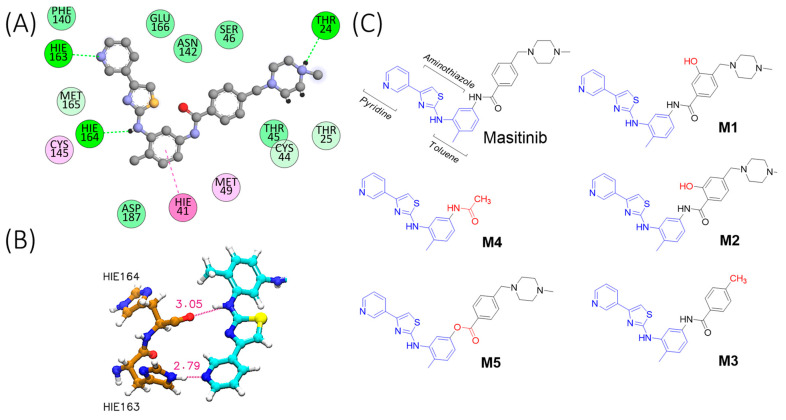
Ligand–protein interactions and chemical structures. (**A**) Interactions between masitinib and M^pro^ residues. The small molecule is shown as grey chemical structure with heteroatoms highlighted in red (Oxygen), blue (Nitrogen) and yellow (Sulphur). The colours of the interactions correspond to π–π stacking (magenta), alkyl–π stacking (pink), hydrogen bonds (bright green), and Van der Waals interactions (green). The interaction maps were prepared using the BIOVIA Discovery Studio Visualizer [17]. (**B**) Schematic representation of key hydrogen bond interactions, displaying masitinib with His^163^ and His^164^. Hydrogen bonds are represented as dotted magenta lines, and the heavy distance (Å) is also shown with the same colour. The key residues His^163^ and His^164^ are shown in brown (Carbon), whereas masitinib (only a fragment, for simplicity) is depicted in cyan (Carbon). Heteroatoms are shown in red (Oxygen), blue (Nitrogen) and yellow (Sulphur). (**C**) Chemical structures of the proposed masitinib derivatives (**M1**–**M5**).

To characterize the M^pro^-masitinib complex dynamics, we began by performing exhaustive, unbiased molecular dynamics simulations. Starting from the complex’s crystallographic structure, it was observed that masitinib binds non-covalently between domains I and II of M^pro^ and blocks the key catalytic residues [6]. Figure 1A shows masitinib’s chemical structure, as well as a schematic representation of the interactions occurring in the masitinib-M^pro^ complex. The nitrogen atom of masitinib’s pyridine ring forms a hydrogen bond with His^163^, while the secondary amine between the toluene and aminothiazole rings forms a hydrogen bond with His^164^ (Figure 1B). Finally, the *N*-methylpiperazine group is outside of the protease binding site and is disordered, displaying no specific interaction with M^pro^ in our model. 

Table 1 provides a compilation and evaluation of the ligand–protein hydrogen bond (H-bond) interactions throughout the dynamics. We report the fraction of time in the unbiased simulations that a given H-bond interaction is sustained based on the following geometrical criteria: a heavy atom distance lower than 3.5 Å and a donor–hydrogen–acceptor angle higher than 140°. Two different protonation states were considered for masitinib: the ligand with its *N*-methylpiperazine group protonated (referred in Table 1 as M(wt)^1^) and the uncharged state (referred as M(wt)^0^). Also, M^pro^ isoforms from both SARS-CoV-2 or HCoV-OC43 were studied. HCoV-OC43 is a human beta-coronavirus that causes the common cold. It was recently shown that HCoV-OC43 is a good biosafety-level 2 (BSL-2) model system to study antiviral drugs against SARS-CoV-2 infection [6]. The two homologous proteins share roughly 50% of sequence identity [16], with the catalytic dyad of Cys-His conserved throughout the two sequences, as they are essential for enzyme activity. Figure S1 shows a comparison between both structures, where the high structural identity is evident (RMSD for the active site main chain is lower than 1.7 Å). 

In the model of M^pro^ from SARS-CoV-2, we find that masitinib in either protonation state can establish highly stable H-bond interactions with His^164^ and His^163^ (Table 1). The His^164^ interaction is formed roughly 90% of the simulation time, while the His^163^ interaction is present more than 50% of the simulation time. Figure S4 from the Supplementary Materials shows the structural properties (heavy atoms distance) as a function of simulation time for these two hydrogen bond interactions in the masitinib-M^pro^ complex. In addition, the *N*-methylpiperazine group displays highly labile interactions with Thr^24^, being present in less than 10% of the simulation period. In the case of M^pro^ from HCoV-OC43, we observe that masitinib in both protonation states can form the same interactions with His^163^ through its piperidine functional group. Additionally, in the HCoV-OC43 model, the secondary amine between the toluene and aminothiazole rings forms hydrogen bonds with Gln^164^. Even though these two interactions are slightly less stable compared to M^pro^ from SARS-CoV-2, they remain formed in the three replicas of each protonation state. Likewise in the complex with M^pro^ from SARS-CoV-2, the *N*-methylpiperazine group displays much labile and less specific interactions with residues as Asn^24^ in the case of M^pro^ from HCoV-OC43. Taken together, these results suggest that masitinib could serve as a platform for the design of broad-spectrum antivirals. We conclude from this analysis that highly stable and specific H-bond interactions are displayed by masitinib with its pyridine and secondary amine groups. These interactions are evident in the two M^pro^ homologs proteins at both protonation states explored here. Furthermore, to probe the importance of these interactions for masitinib binding, we explored a third protonation state where the masitinib’s pyridine ring is also protonated in addition to the *N*-methylpiperazine group (ligand net charge equal to +2). In this way, the H-bond interaction with His^163^ is disturbed. Figure S3 compares the RMSD for the ligand heavy atoms as a function of the simulation time in three independent replicas for masitinib with net charge equal to +1 and +2. From these simulations, the importance of this interaction becomes evident; His^163^ works as a key anchoring point of the inhibitor. Its perturbation leads to ligand unbinding within nanoseconds.

We also estimated the root mean square fluctuations (RMSF) for the ligand heavy atoms in the aforementioned systems. As shown in Figure S2, there are low thermal fluctuations for masitinib’s pyridine, aminothiazole, and hydrophobic toluene rings, which interact by means of π–π-stacking with the catalytic His^41^. In contrast, the benzoyl and *N*-methylpiperazine groups exhibit increased thermal fluctuations, with the highest values for the *N*-methylpiperazine group. These results, in conjunction with the analysis of the H-bond interactions, highlight the importance of masitinib’s pyridine, aminothiazole, and toluene rings for M^pro^ inhibition. Based on these results, we conclude that the benzoyl and N-methylpiperazine groups of masitinib do not form specific interactions. 

Based on the masitinib-M^pro^ binding interactions outlined above, we designed a small library of masitinib derivatives. We hypothesized that the lead structures shown in Figure 1C might have a higher binding affinity towards the SARS-CoV-2 M^pro^ protein by altering the low affinity benzoyl and N-methylpiperazine functionalities. **M1** and **M2** introduce an H-bond donor and acceptor pair in the form of a hydroxyl group affixed to the benzoyl ring, whereas the **M3** derivative lacks the final *N*-methylpiperazine group altogether. **M4** keeps the minimal fragment responsible for masitinib inhibition (the pyridine, aminothiazole, and hydrophobic toluene rings) but lacks both the benzoyl and *N*-methylpiperazine groups. Finally, in **M5**, an ester linkage is employed instead of the masitinib’s amide linkage to explore the effect of suppressing the H-bond donor capability at this position. From our previous analysis of the non-covalent interactions taking place in the masitinib-M^pro^ complex, no specific interactions were expected to occur at this site.

The binding free energy of a molecular complex provides a rigorous thermodynamic measure of the degree of affinity of two molecules for each other. Thus, we have estimated and compared the absolute binding free energy for **M1** to **M4** by means of two different methods: linear interaction energy (LIE-D) [18] and thermodynamic integration (TI). It is important to mention that **M5** was the only analogue that exhibited high RMSD values (Figure S6). Therefore, we did not perform the absolute binding calculations with the most demanding TI method for this compound on account of the high uncertainty with respect to its binding mode. 

Table 2 provides the absolute binding free energy calculations for masitinib and its derivatives. The inhibition constant, K_i_ (inhibitor concentration needed to occupy half of the enzyme active sites), for masitinib activity on M^pro^ was determined to be 2.6 μM [6]. The absolute binding free energy for masitinib determined here by means of thermodynamic integration is in quantitative agreement (within its uncertainty) with the experimental measurements. We note that all the proposed masitinib analogues (**M1**–**M5**) were predicted to form a thermodynamically stable complex with M^pro^, as illustrated by the low binding free energies reported in Table 2. The **M1** to **M3** analogues exhibit a similar performance to that of masitinib, with no quantitative differences with the more rigorous TI calculations. In contrast, based on the TI calculations and LIE-D, **M4** shows the lowest free energy values (in absolute terms), and it could be expected to exhibit a lower M^pro^ inhibition. Finally, based on the LIE-D results, a lower performance is also expected for **M5** compared to masitinib.

Table S1 lists the ligand–protein hydrogen bond interactions (H-bond) throughout our dynamic runs for **M1**–**M5**. In all cases, the reported fraction for each interaction corresponds to the average from three independent replicas. From these data, it is evident that the **M3** and **M4** analogues are able to maintain the key anchoring interactions with His^164^ and His^163^. Also, **M2** exhibits a new H-bond interaction through its hydroxyl group with Cys^44^ that is stable over more than 60% of the runtime. The **M5** analogue exhibits a highly dynamic complex, with several weak interactions taking place. The deep reduction in the lifetime for the key anchoring interactions (H-bond with His^164^ and His^163^) is apparent in **M5**, where these contacts are broken more than 70% of the runtime. Figure S5 depicts 2D contact maps for all interactions between M^pro^ and the **M1**–**M4** derivatives. The 2D contact map for **M5** is not shown due to its highly dynamic nature and the uncertainty associated with its binding mode. Having successfully predicted the interactions between our masitinib derivatives and M^pro^, we synthesized these compounds to characterize their activity in vitro and to validate our computational models.

### 2.2. Compound Synthesis

**Figure 2:**
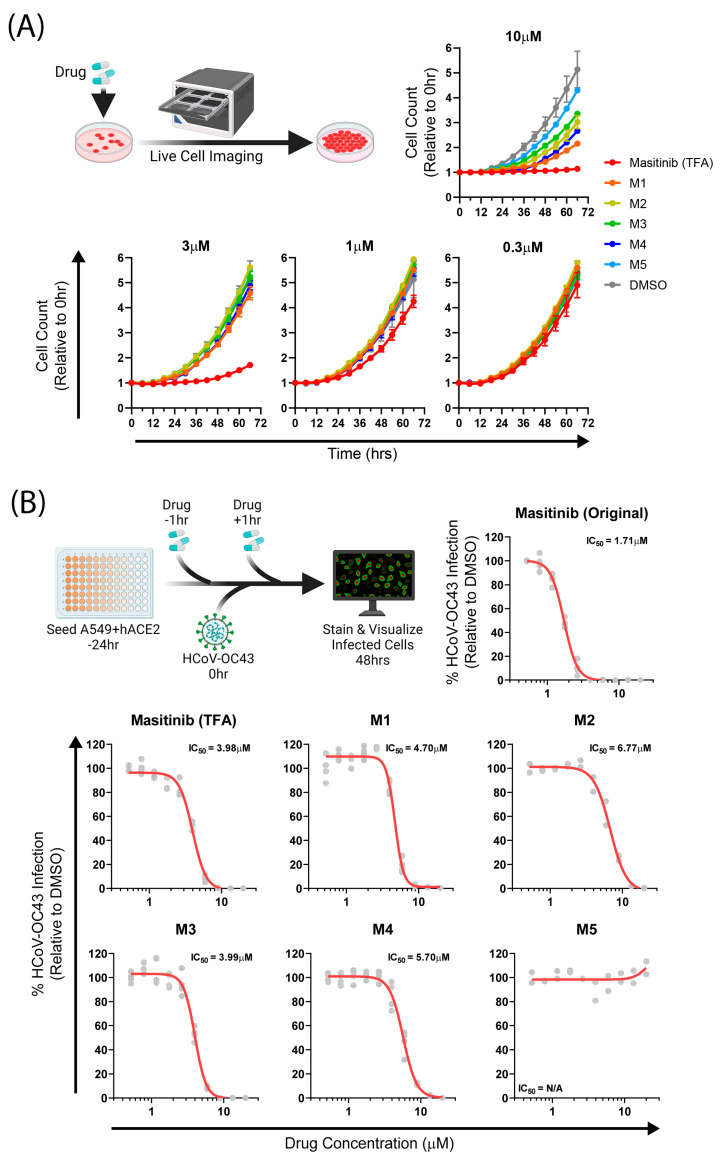

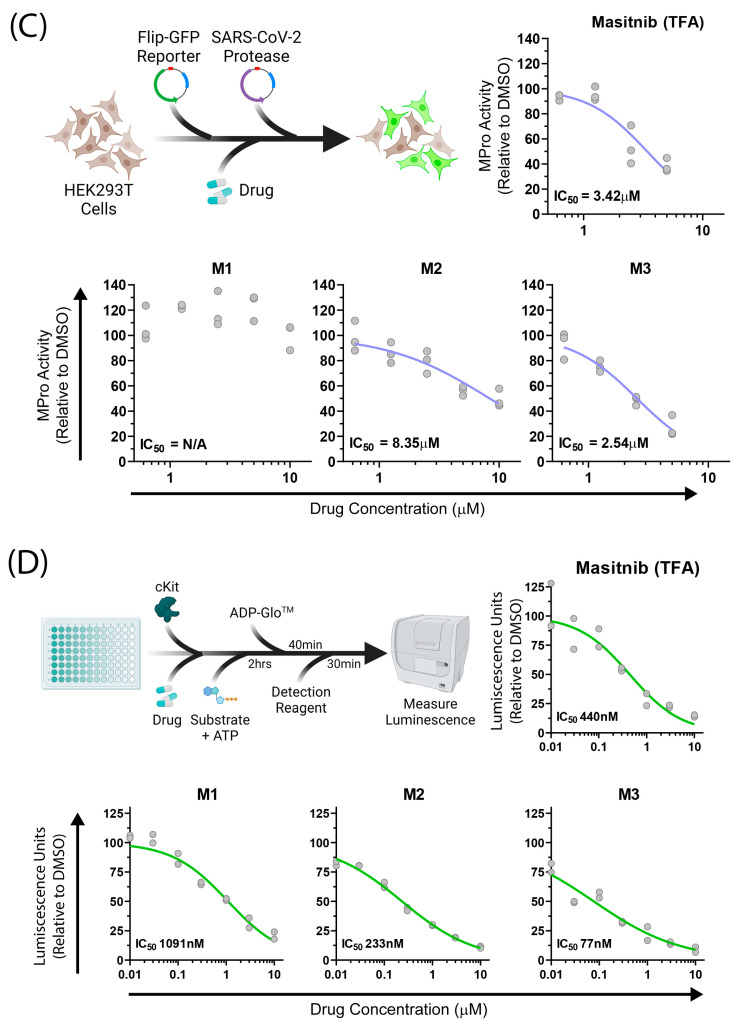
Mechanisms of masitinib analogue antiviral activity. (**A**) A549+H2BmRuby cells were seeded at low confluency and imaged every 6 h. Cell count was quantified using H2BmRuby fluorescence and normalized to 0 h timepoint. (**B**) A549+hACE2 cells treated with masitinib and its analogues were infected with HCoV-OC43, and virus-infected cells were stained at 48 hpi. (**C**) HEK-293 cells were transfected with Mpro-, Flip-GFP-, and TagBFP2-expressing vectors and treated with various concentration of masitinib and its analogues. After 24 h, GFP expression was quantified and normalized to TagBFP2 expression for each condition. (**D**) Masitinib and its analogues were incubated with c-Kit enzyme, and substrate processivity was assessed using ATP depletion by luminescence activity. Each grey point indicates a replicate (**A**) *n* = 2 in technical triplicates; (**B**) *n* = 2–3 in technical duplicates; (**C**) *n* = 2, each in technical duplicates; and (**D**) *n* = 3. Experimental outlines were designed on BioRender.

The **M1**–**M5** derivatives were synthesized from 2,4-diaminotoluene or 2-amino-4-methoxytoluene using a modified literature preparation method for the synthesis of masitinib [19]. Full synthetic schemes are provided in the Supplementary Information (Schemes S1 and S2). After successfully synthesizing all compounds, the parent masitinib compound and **M1**–**M5** were purified using preparative HPLC as trifluoroacetate (TFA) salts, freeze-dried, and dissolved in DMSO immediately prior to experimentation. ^1^H-NMR, ^13^C-NMR, and ESI-MS were used to confirm the identity of the novel compounds. A commercially prepared version of masitinib was also compared to our masitinib-TFA produced in house to confirm that the efficacy of the prepared compounds is comparable to that of commercially sourced samples (Figure 2B). Having successfully synthesized masitinib and **M1**–**M5**, we proceeded to probe their binding affinity and anti-viral efficacy in vitro.

### 2.3. Biological Assays

We used several in vitro cytotoxicity and infection models to probe the activity of our analogues. In clinical trials, masitinib treatment has demonstrated cardiac toxicity and dermatological disorders in some patients [20,21]. We therefore began by assessing the in vitro cytotoxicity profiles of masitinib and its derivatives. A549 human lung adenocarcinoma cells were plated at low density and treated with the compounds, followed by live cell imaging to monitor cell growth over time. At the 10 µM compound dose, masitinib (TFA) completely inhibited cell growth, whereas all derivatives allowed cell proliferation, albeit at reduced levels compared to DMSO. Derivatives **M5** and **M1** had the least and highest inhibitory effects on cell proliferation, respectively. At a 3 µM dose, all derivatives yielded cell proliferation similar to that of DMSO. However, masitinib (TFA) at 3 µM still inhibited cell division, suggesting that it is a potent cytostatic compound and our derivatives are less toxic. All doses lower than 3 µM had similar cell growth kinetics to DMSO (Figure 2A, Table 3). 

SARS-CoV-2 and HCoV-OC43 are members of the betacoronavirus genus and share a high homology of the M^pro^ protease [22]. Since SARS-CoV-2 studies must be performed in high containment biosafety level-3 (BSL-3) facilities, many previous studies have relied on HCoV-OC43 as an effective model for SARS-CoV-2 antiviral screening in a BSL-2 environment [6,23]. Here, A549+hACE2 cells were infected with HCoV-OC43 in the presence of masitinib or its analogues for 48 h, followed by staining and quantification of virus-infected cells. We initially performed a comparative antiviral assay between commercially available and in-house synthesized masitinib to determine if our synthesis protocol had any effects on antiviral potency. Commercial masitinib (Original) had an IC_50_ of 1.71 µM, whereas in-house synthesized masitinib (TFA) had an IC_50_ of 3.98 µM, suggesting a slightly lower potency of the in-house synthesized version relative to the commercial compound. This change in potency could result from differences in the counterion between the isolated and commercial forms. Of the 3 masitinib analogues we synthesized, **M3** had an IC_50_ of 3.99 µM, while **M1** had an IC50 of 4.70µM, both similar to that of masitinib (TFA). **M2** and **M4**, however, had higher IC_50_s than masitinib (TFA), namely 6.77 µM and 5.70 µM, respectively. **M5**, on the other hand, had no measurable antiviral activity (Figure 2B). Derivatives **M1**, **M2**, and **M3** had the most similar antiviral potencies to masitinib (TFA) and were selected for further characterization. 

To delineate the mechanism of antiviral activity, we first assessed the derivatives’ inhibitory potential on the SARS-CoV-2 M^pro^ enzyme. A cell-based protease assay was performed to assess protease inhibition in a complex cellular environment that accounts for the potential of altered cellular target binding of masitinib derivatives. HEK-293T cells were transfected with plasmids expressing the flip-GFP reporter, SARS-CoV-2 M^pro^, and TagBFP2 as a transfection control. Transfected cells were treated with compounds and assessed for GFP expression as a proxy for M^pro^ activity at 24 h post transfection. **M3** had a lower approximate IC_50_ compared to masitinib (TFA), while **M2** had a higher approximate IC_50_, following the trend observed in the HCoV-OC43 antiviral assay (Figure 2B,C). Interestingly, **M1** did not show inhibition of M^pro^ activity, suggesting that HCoV-OC43 antiviral activity by **M1** was likely mediated by host targeting. 

Masitinib’s primary known host targets are c-Kit kinase and PDGFA/B, all of which are expressed at very low levels in A549 cells (EMBL Expression Atlas), indicating that other potential targets of masitinib are responsible for the observed cytostatic effects. Nevertheless, c-Kit is expressed in various human tissues including the brain, respiratory system, and skeletal muscles. To assess the altered binding potential of the masitinib derivatives to host targets, we performed a luminescence-based in vitro c-Kit kinase assay to assess the host target inhibitory capacity of the masitinib analogues. The compounds were incubated with the c-Kit enzyme, followed by the addition of the c-Kit specific substrate. The luciferase detection of ADP accumulation as a result of ATP utilization was used as an indirect measure of kinase activity. Compared to masitinib (TFA), **M1** had lower activity against c-Kit, whereas **M2** and **M3** had a higher activity against c-Kit (Figure 2D). Given the low c-Kit expression in A549 cells (EMBL Expression Atlas), the antiviral activity of **M3** and **M2** is likely mediated by direct inhibition of M^pro^. In contrast, **M1** had highly attenuated c-Kit inhibition and no M^pro^ restriction but still possessed antiviral activity similar to that of masitinib (TFA), suggesting a c-Kit-independent host target important for HCoV-OC43 virus replication. Importantly, all masitinib derivatives had diminished cytostatic effects, as was initially strategized. 

## 3. Discussion and Conclusions

As indicated earlier, masitinib (a tyrosine kinase inhibitor) has potential as a broad-spectrum inhibitor for human coronaviruses, including SARS-CoV-2 and its variants [6]. However, masitinib treatment may lead to cardiac toxicity and dermatological disorders in a clinical setting in some patients [20,21]. By relying on advanced-sampling simulations, we developed a molecular-level understanding of masitinib’s mechanism of M^pro^ inhibition. Based on an analysis of the key interactions of Masitinb with M^pro^, we then designed and tested several new derivatives with higher efficiency and lower cytotoxicity. 

At a molecular level, masitinib was shown to act as a competitive inhibitor for the SARS-CoV-2 main protease (M^pro^ or 3CLpro). The masitinib-M^pro^ complex’s crystallographic structure indicates that masitinib binds non-covalently between domains I and II of M^pro^ and blocks the key catalytic residues Cys145 and His^41^ [6]. Our molecular simulations indicate that highly stable and specific H-bond interactions are displayed by masitinib with its pyridine and aminothiazole rings. The nitrogen atom of the pyridine ring forms a H-bond with His^163^ and the masitinib aminothiazole ring forms another H-bond with His^164^ (through its amine functional group). Of importance, His^163^ is a key anchoring point of the inhibitor, and its perturbation leads to ligand debinding within nanoseconds. In contrast, the terminal *N*-methylpiperazine group displays highly labile interactions with Thr^24^. Finally, masitinib’s aminothiazole and toluene rings interact through π–π interactions with the catalytic His^41^, enhancing specific non-covalent interactions with its molecular target, M^pro^. 

Based on these results, we hypothesized that higher potency could be achieved by altering the low-affinity benzoyl and *N*-methylpiperazine functionalities. We proposed a small library of masitinib derivatives and examined their efficacy in simulations and experiments. We used in vitro cytotoxicity and infection models to probe the activity of the proposed **M1** to **M5** analogues. All masitinib derivatives proposed here exhibited reduced cytotoxic properties compared to masitinib; compounds **M5** and **M1** had the smallest and highest inhibitory effects on cell division, respectively. Compounds **M1** and **M2** exhibited lower antiviral activity compared to masitinib, showing that the introduction of an H-bond donor and acceptor functional group (hydroxyl) in the benzoyl ring is not an effective strategy to increase the low affinity of this unit. Moreover, **M1** had no inhibition of M^pro^ activity. One reason for the discrepancy between inhibitory assays and simulations could be an incorrect initial assignment of the binding mode of **M1**. In contrast, a deep reduction in the lifetime of the key anchoring interactions (H-bond with His^164^ and His^163^) was evident in simulations of the **M5** derivative. This result agrees with antiviral assays where **M5** showed no measurable activity. In this regard, it is also important to highlight that ester functionalities can undergo degradation in cellular environments—a phenomenon that has been observed in various experimental settings. As an example, in the context of drug delivery or prodrug design, ester linkages are deliberately chosen for their susceptibility to enzymatic or chemical cleavage by esterase [24]. In this sense, the metabolic capacity of the A549 cell line based on esterase activities was recently characterized [25]. The main findings of that study were that esterase enzymes (CES1 and CES2) are present in these cell lines, regardless of their pulmonary origin [25]. Based on that report, we speculate that the hydrolyzation of **M5** is also probable in our cellular assay. Finally, the **M3** derivative, which lacks the final *N*-methylpiperazine group, exhibited an antiviral potency comparable to that of masitinib, and a higher inhibitory potency of the SARS-CoV-2 M^pro^ enzyme. These results suggest that **M3** is a promising platform for the design of new broad-spectrum antivirals. 

Normalization of affinity with respect to molecular size is recommended in drug discovery. Ligand efficiency (LE) is a value that expresses the binding energy of a small molecule normalized by the molecule’s size; typically, it is calculated by scaling affinity by the number of heavy atoms (non-hydrogen atoms). The most used method of calculating LE is to divide the Gibbs free energy of binding (Δ*G*) by the number of heavy atoms (*N*) as follows: LE *=* Δ*G*/*N*. Here, we have estimated the LE from IC_50_ values of the phenotypic antiviral screening. Then, equating IC_50_ with *K*_i_ and relying on the thermodynamic Gibbs free energy equation, Δ*G* = −RTlnK_i_, the LE can be expressed as LE = 1.37(pIC_50_)/*N* [15], where pIC50 is the −log_10_(IC_50_). Thus, the **M4** derivative, which maintains the minimal fragment responsible for most of the non-covalent interactions (the pyridine, aminothiazole, and hydrophobic toluene rings), exhibited an antiviral activity of the same order of magnitude as masitinib, and this is translated into an improvement in the LE of roughly 50% for **M4** (LE = 0.252) compared to masitinib (LE = 0.167). Similarly, an increase of roughly 25% is observed for **M3** (LE = 0.208) compared to masitinib (LE = 0.167). Considering the lower cytotoxicity, protease-dependent antiviral activity, and overall small size, we consider **M3** to be a good starting option for further lead optimization cycles. Follow-up studies into antiviral mechanisms of action of other small derivatives with reduced cytotoxicity, such as **M4**, are currently being assessed.

## 4. Material and Methods

Modeling of M^pro^, Masitinib, and derivatives:

The three-dimensional structures of the different molecules under evaluation were generated by means of the program UCSF Chimera [26]. The initial configuration of the M^pro^ (SARS-CoV-2)-masitinib complex was taken from the Protein Data Bank (PDB code: 7JU7) [6]. The M^pro^ structure from HCoV-OC43 is the homology model generated with Modeller based on the HKU1 M^pro^ structure (PDB code:3D23) [27].

### 4.1. Molecular Docking 

Molecular docking simulations of M^pro^ were performed with the Autodock 4.2 simulation package [28]. Crystallization water molecules and small organic molecules were removed from the receptor binding site. The resulting structure was minimized; then, Gasteiger charges were added; and finally, nonpolar hydrogen atoms were removed with the Autodock Tools (ADT) program [28]. A cluster analysis based on heavy atom root mean square deviation (RMSD) was performed over the resulting docked conformations for each ligand. The lowest docking energy conformation of each cluster was considered the most favourable orientation. 

### 4.2. Molecular Dynamics

The AMBER 20 simulation package [29] was used to perform atomistic molecular dynamics simulations. The ligand–protein complexes’ parameters were derived from the ff14SB force field [30] (proteins) and the general amber force field GAFF [31] (small molecules). The charges for the ligands were estimated with an antechamber using the AM1-BCC charge model [32]. The Leap software (distributed as part of AmberTools20 [29]) was used to add hydrogens to the protein. In addition, we chose the protonation state of critical His^163^ to preserve the hydrogen bond interaction, as found in the complex crystal structure. Thus, both histidine residues (His163 and His164) were protonated at the epsilon position. In all cases, an octahedral box with ~12,000 TIP3P water molecules and ~4 Cl^−^ ions was used. During equilibration, a Langevin thermostat was used to increase the temperature of the system gradually from 0 to 300 K, over 500 ps. Equilibration/production runs were performed in a constant pressure ensemble following previously reported methods [33]. A Particle Mesh Ewald method (12 Å cutoff) was used to evaluate long-range electrostatic interactions. In all cases, 3 replicas of at least 600 ns were considered, each one giving a cumulative simulation time of more than 12 µs (5 ligands plus 2 protonation states for masitinib in two different contexts). 

### 4.3. TI Calculations

The absolute free energy for ligand binding was estimated as follows:

ΔG_Absolute_ = ΔG_Ligand_ − ΔG_RL_ − ΔG_res_

Thus, the binding absolute free energy ΔG_Absolute_ is determined as the difference between the free energy change in ligand annihilation in water (ΔG_Ligand_) and ligand annihilation in the M^pro^ protein (ΔG_RL_). Additionally, as we imposed a single virtual bond between the protein and the small molecule, an additional correction term was considered, and ΔG_res_ represents the free energy correction due to the imposed restraints in the simulation. 

We implemented a one-step annihilation protocol with soft core potentials. Four independent runs were performed, each starting from the equilibrated ligand position obtained from previous simulations. Eleven equally spaced windows were selected at lambda values equal to 0.0; 0.1; 0.2; 0.3; 0.4; 0.5; 0.6; 0.7; 0.8; 0.9; and 1.0. In all cases, a 50 ns simulation time per window was considered. The imposed restraints added a correction to the free energy, given by

$$∆Gres=-kbTln\;\left\lceil \frac{8\pi^{2}V{}^{\circ}{Kr}^{1/2}}{{\left(2\pi kbT\right)}^{1/2}}\right\rceil$$

where *V*° is the standard state volume and *Kr* is the force constant, in this case, 10 kcal/mol. 

### 4.4. LIE-D Calculations 

In the linear interaction energy (LIE) method, the binding free energy is expressed as follows:

ΔG_bind_ = βΔ<V*_l_*_-*s*_>^el^ + αΔ<V*_l_*_-*s*_>^vdw^ + γ

where <V*_l-s_*>^el^ and <V*_l-s_*>^vdw^ are ensemble averages corresponding to the non-bonded electrostatic and van der Waals interactions of the ligand (*l*) with its environment (*s*), and Δ means the average energy change from transferring the ligand from the aqueous solution to the hydrophobic binding site of the protein (in this case, M^pro^). The coefficients α and β are scaling factors for these energy terms. Here, we used the values α = 0.18 and β = 0.50. The empirical constant γ was estimated using the linear fitting parameters in Figure 1 of Miranda et al. [18].

The <V*_l_*_-*s*_>^el^ and <V*_l_*_-*s*_>^vdw^ ensemble averages were estimated using CPPTRAJ [34], where the non-bonded energy (van der Waals and electrostatic interactions) was calculated between the ligand and the rest of the system (protein, ions, and water). The results for each system in each replicate were averaged over 1500 frames from the last 500 ns. For the ligand in aqueous solution, we considered 1000 frames from one single replica of 100 ns.

### 4.5. Hydrogen Bond Analysis

Hydrogen bonds were analysed with CPPTRAJ [34], where the cutoffs for the distance between heavy atoms and the associated angle were set to 3.5 Å and 40° (to account for directionality), respectively. 

Biological assays.

### 4.6. Cells

A549-hACE2 (human lung carcinoma cell line expressing human angiotensin-converting enzyme 2) was obtained through BEI Resources, NIAID (Cat #NR-53821). The A549 and HEK293T cells were originally obtained from ATCC. All cell lines were cultured in DMEM media that was supplemented with 10% foetal bovine serum (FBS). 

### 4.7. Viruses

HCoV-OC43 (ATCC, Cat# VR-1558) was purchased from ATCC and propagated in A549 cells as previously described [6].

### 4.8. HCoV-OC43 Infections

HCoV-OC43 infections were performed on A549-hACE2 cells at 33 °C. A549-hACE2 cells were infected with HCoV-OC43 at an MOI of 0.1 for 1 h, followed by the addition of compound/DMSO diluted in DMEM supplemented with 5% FBS and incubated for an additional 48 h. After cell fixation with 4% paraformaldehyde and blocking/permeabilization, cells were incubated with subsequent rounds of primary HCoV-OC43 antibody (1:1000 dilution, MilliporeSigma Cat# MAB9013, Darmstadt, Germany) and secondary CF^®^647 antibody (1:1000, Biotium Cat#20047, Fremont, CA, USA). All antibodies were diluted in blocking/permeabilization buffer (3% Bovine Serum Albumin/PBS/0.1% Triton X-100) and incubated for 1 h at room temperature. Cell nuclei were stained with Hoecsht 33342 (1:10,000, Invitrogen Cat# H3570, Waltham, MA, USA), and images were captured using a Nikon Eclipse Ti2 microscope. Image analysis was performed using CellProfiler version 4.2.5 (Broad Institute). HCoV-OC43 infection was calculated as the cell area with positive HCoV-OC43 staining relative to the total cell area. All values were normalized to DMSO-treated wells.

### 4.9. Compound Toxicity Assays

A549 cells were seeded in 96-well plates at 2000 cells/well with media supplemented with various doses of each compound. Cells were monitored and imaged every 6 h on an Incucyte^®^ S3 Live-Cell Analysis System (Sartorius, Göttingen, Germany). Cell confluency was measured using the automated workflow on the Incucyte^®^ software version 2020B.

### 4.10. SARS-CoV-2 M^pro^ Activity

HEK293T cells were transfected with 100 ng of total plasmid per 96 well. Flip-GFP:M^pro^:TagBFP2 was used in a ratio of 1:0.8:3.2. TransIT-LT1 (Mirus, Cat#2300) transfection reagent was incubated with a plasmid cocktail for 15 min at room temperature, at a ratio of 0.3 μL of the LT1:100 ng plasmid. The transfection mixture was added to the cells, immediately followed by the addition of the drug dilution, and incubated at 37 °C for 24 h. Cells were imaged for GFP and TagBFP2 expression using a Nikon Eclipse Ti2 microscope. Image analysis was performed using CellProfiler version 4.2.5 (Broad Institute). GFP expression was normalized to TagBFP2 expression. Drug doses whose TagBFP2 expression was less than 70% of DMSO control wells were excluded from the analysis. Flip-GFP and Mpro plasmids were a kind gift from Dr. Nicholas Heaton, Duke University.

Organic chemistry.

### 4.11. Materials

All chemicals unless otherwise noted were obtained from Sigma Aldrich or Thermo Fisher and used without further purification. Methyl 4-(bromomethyl)-2-methoxybenzoate was purchased from AA Blocks. 2-Bromo-1-pyridin-3-ylethan-1-one was purchased from Matrix Scientific. Masitinib was purchased from Selleck Chemicals. When denoted as dry, DCM was refluxed over CaH_2_ and distilled prior to use. 

### 4.12. Instrumentation

Preparative HPLC was conducted on a Gilson Gx271 system equipped with a 150 × 21.2 mm Luna PREP-C18(2) 100 Å column. ESI-MS characterization was conducted on an Agilent 6135 quadrupole LC/MS system using 1:1 ACN:H_2_O + 0.1% TFA. ^1^H and ^13^C NMR spectra were taken at 25 °C on a 400 MHz Bruker DRX instrument equipped with a BBO probe using Topspin 1.3 or at 25 °C or 100 °C on a 500 MHz Bruker Avance-II+ spectrometer equipped with a QNP probe using Topspin 2.1. NMR spectra were analysed using MestreNova. Spectra are referenced to the residual protonated solvent peak for ^1^H NMR (DMSO-*d*_6_ = 2.50 ppm, CDCl_3_ = 7.26 ppm) and ^13^C {^1^H} NMR (CDCl_3_ = 77.16 ppm, DMSO-*d*_6_ = 39.52 ppm).

### 4.13. Synthetic Procedures and Characterization

Scheme S1 from the Supplementary Materials shows the synthesis of masitinib, **M1**, **M2**, and **M3**, adapted from reference [19]. The key chemical transformation involves amidation of **6** with the esters **7a**–**d** using AlMe_3_, followed by deprotection with BBr_3_ to form compounds with modifications at the X_1_, X_2_, and R sites.

Synthesis of 6-methyl-N^1^-(4-(pyridin-3-yl)thiazol-2-yl)benzene-1,3-diamine (**6**):


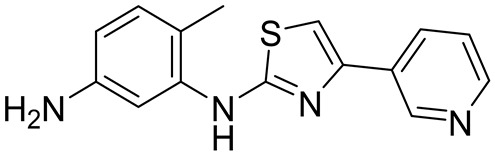


The title compound was prepared following a modified literature procedure^S1^ starting from 2,4-diaminotoluene. ^1^H NMR (500 MHz, DMSO *d*_6_, 298 K): 9.21 (s, 1H), 9.09 (d, 1H), 8.48 (dd, 1H), 8.21 (dt, 1H), 7.41 (dd, 1H), 7.37 (s, 1H), 7.11 (d, 1H), 6.87 (d, 1H), 6.30 (dd, 1H), 4.94 (br s, 2H), 2.11 (s, 3H). ^13^C {^1^H} NMR (126 MHz, DMSO-*d*_6_, 298 K): 166.7, 148.2, 147.4, 147.2, 147.0, 139.6, 132.8, 131.0, 130.4, 123.7, 116.6, 110.4, 107.9, 103.9, 17.1. ESI-MS: M+H^+^ = 283.1 expected, 283.0 observed.

General procedure for the synthesis of **7a**–**c**:


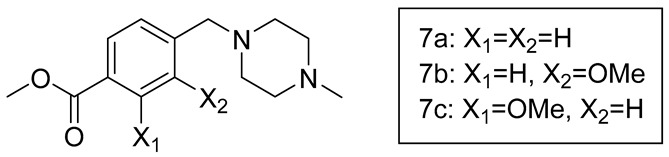


The synthesis of **7b**–**c** was adapted from a reported synthesis of **7a** [19]. We added to a round bottom flask 1-methylpiperazine (1.1 eq), triethylamine (2.0 eq), ethanol, and a stir bar. The flask was fitted with an addition funnel, sealed under argon, and cooled to 0 °C in an ice bath. Methyl 4-(bromomethyl)benzoate or a derivative (1.0 eq) dissolved in ethanol was added via a syringe to the addition funnel and added dropwise over 15 min to the reaction under rapid stirring. After complete addition, the vessel was warmed to room temperature and was stirred for 18 h. After complete consumption of the start material was confirmed using LC-MS, we removed the solvent at reduced pressure and the crude product was partitioned between 30 mL of H_2_O and 70 mL of DCM. The organic phase was collected, dried over MgSO_4_, loaded onto silica, and purified using silica gel chromatography (50–100% EtOAc in hexanes + 2% TEA). The product fractions were pooled, and the solvent was removed under reduced pressure to obtain the products as yellow oils.

Methyl 4-((4-methylpiperazin-1-yl)methyl)benzoate: ^1^H-NMR (400 MHz, CDCl_3,_ 298 K): 7.98 (d, 2H), 7.40 (d, 2H), 3.91 (s, 3H), 3.55 (s, 2H), 2.75–2.33 (br s, 8H), 2.29 (s, 3H). ^13^C{^1^H} NMR (298 K, CDCl_3_, 101 MHz): 167.08, 143.84, 129.57, 128.97, 128.94, 62.62, 55.09, 53.13, 52.05, 46.00. ESI-MS: M + H^+^ = 249.1 expected, 249.0 observed.
Methyl 2-methoxy-4-((4-methylpiperazin-1-yl)methyl)benzoate: ^1^H-NMR (400 MHz, DMSO-*d*_6_, 298 K): 7.61 (d, 1H), 7.07 (s, 1H), 6.96 (d, 1H), 3.82 (s, 3H), 3.76 (s, 3H), 3.50 (s, 2H), 2.48–2.22 (br s, 8H), 2.16 (s, 3H). ^13^C{^1^H} NMR (293 K, CDCl_3_, 101 MHz): 166.62, 159.33, 145.01, 131.63, 120.62, 118.56, 112.34, 62.75, 56.05, 55.13, 53.18, 51.94, 46.04. ESI-MS: M + H^+^ = 279.2 expected, 279.0 observed.
Methyl 3-methoxy-4-((4-methylpiperazin-1-yl)methyl)benzoate: ^1^H-NMR (400 MHz, CDCl_3,_ 298 K): 7.60 (d, 1H), 7.49 (s, 1H), 7.43 (d, 1H), 3.89 (s, 3H), 3.85 (s, 3H), 3.57 (s, 2H), 2.60–2.38 (br s, 8H), 2.27 (s, 3H). ^13^C{^1^H} NMR (101 MHz, CDCl_3_, 298 K): 167.10, 157.53, 131.99, 129.78, 129.73, 121.81, 111.01, 55.80, 55.57, 55.15, 53.16, 52.10, 46.01. ESI-MS: M + H^+^ = 279.2 expected, 279.1 observed.

General procedure for the synthesis of amides **1**, **8a**–**b**, and **M3**:


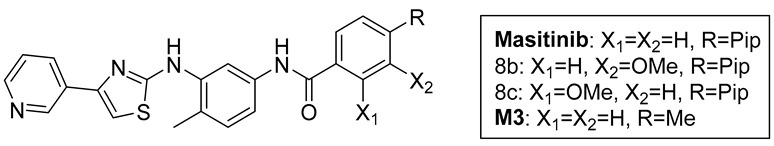


A Schlenk flask was charged with **6** (1.0 eq.) and dry CH_2_Cl_2_ (concentration ca. 0.1 M) under an argon atmosphere. The vessel was cooled to 0 °C, and AlMe_3_ (4 eq. as 2M solution in toluene) was added dropwise. We warmed the reaction mixture to RT and stirred it for 30 min. After this time, the corresponding ester (**7a**–**d**) was added (1.0 eq.) and the reaction mixture was subsequently heated to reflux and was stirred. After 12 h, the mixture was cooled to 0 °C, and a 2M aqueous NaOH solution (excess, >50 eq.) was added. We extracted the mixture with CH_2_Cl_2_ (2 × mL) and dried organic layers with MgSO_4_, which was then filtered, and the solvent removed under reduced pressure to leave a yellow residue. Crude masitinib and **M3** were each dissolved in 3:1 H_2_O:ACN + 0.1% TFA and purified via preparative HPLC using a 5–60% gradient of ACN in H_2_O + 0.1% TFA. Product-containing fractions were confirmed using ESI-MS, pooled, and freeze-dried to obtain each derivative as yellow powders. **8b** and **8c** were used in the next reaction without further purification.

Procedure for deprotection of **8b**–**c** to make **M1** and **M2**:


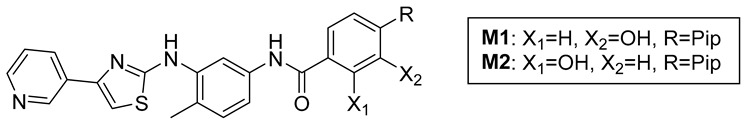


A Schlenk flask was charged with crude **8b**–**c** and dry CH_2_Cl_2_ (5 mL) under an inert atmosphere. Each reaction was cooled to 0 °C, and BBr_3_ (10–20 eq.) was added dropwise. Each reaction mixture was stirred for 12 h at RT. At the end of this procedure, a H_2_O/MeOH solution (10 mL, 1:1 *w*/*v*) was added to each reaction, each of which was stirred for 6 h at RT. The mixture was extracted with EtOAc (2 × 10 mL). We combined the organic layers and washed them with brine (2 × 10 mL), and the solvent was subsequently removed under reduced pressure. The resulting residues were each dissolved in 3:1 H_2_O:ACN + 0.1% TFA and purified via preparative HPLC using a 5–60% gradient of ACN in H_2_O + 0.1% TFA. Product-containing fractions were confirmed using ESI-MS, pooled, and freeze-dried to obtain purified **M1** and **M2** as yellow powders.

Masitinib trifluoroacetate salt: Following the general procedure above, **6** (115 mg, 407 µmol), AlMe_3_ (2M in toluene, 0.82 mL, 1.63 mmol), and **7a** (101 mg, 407 µmol) were reacted to prepare masitinib (85.1 mg) in a 42% yield. NMR spectra matched with literature precedent [19]. ^1^H NMR (500 MHz, DMSO-*d*_6_, 373 K): 9.76 (br s, 1H), 9.10 (d, *J* = 1.5 Hz, 1H), 8.48 (dd, *J* = 4.7, 1.6 Hz, 1H), 8.32 (d, *J* = 2.1 Hz, 1H), 8.25 (dt, *J* = 8.3, 2.1 Hz, 1H), 7.97 (d, *J* = 8.2 Hz, 2H), 7.47 (d, *J* = 8.3 Hz, 2H), 7.40 (m, 2H), 7.29 (s, 1H), 7.20 (d, *J* = 8.3 Hz, 1H), 3.75 (br s, 2H), 3.21 (br s, 4H), 2.79 (br s, 3H), 2.75 (br s, 4H), 2.30 (s, 3H). ESI-MS: M + H^+^ = 499.2 expected, 499.1 observed.
**M1** trifluoroacetate salt: Following the general procedure, **6** (180 mg, 637 µmol), AlMe_3_ (2M in toluene, 0.82 mL, 1.63 mmol), and **7b** (177 mg, 637 µmol) were reacted to prepare crude **8b** (crude yield 226 mg, 67%). A Schlenk flask was charged with a portion of crude **8b** (70 mg, 132 µmol) and reacted with BBr_3_ (0.43 g, 0.16 mL, 1.70 mmol) according to the general procedure to obtain **M1** as a yellow solid (81 mg, 24% over two steps). ^1^H NMR (400 MHz, DMSO-*d*_6_, 293 K): 9.75 (br s, 1H), 9.14 (br s, 1H), 9.24 (d, *J* = 1.7 Hz, 1H), 8.57 (m, 1H), 8.35 (d, *J* = 7.7 Hz, 1H), 8.20 (s, 1H), 7.49 (dd, *J* = 5.2, 2.6 Hz, 1H), 7.44 (s, 2H), 7.43 (s, 1H), 7.34 (d, *J* = 7.6 Hz, 1H), 7.20 (d, *J* = 8.2 Hz, 1H), 3.85 (br s, 2H) 3.24 (br s, 4H), 2.89 (br s, 4H), 2.77 (s, 3H), 2.29 (s, 3H). ^13^C {^1^H} NMR (DMSO-*d*_6_, 126 MHz, 298 K): 166.3, 165.3, 158.2 (q, *J* = 34 Hz), 156.6, 156.3, 145.9, 145.2, 144.1, 139.0, 137.6, 137.5, 136.6, 136.2, 131.5, 130.5, 125.0, 124.2, 124.1, 118.2, 116.2 (q, *J* = 294 Hz), 115.7, 115.0, 114.9, 113.2, 106.3, 51.9, 48.6, 45.1, 42.9, 17.6. ESI-MS: M+H^+^ = 515.2 expected, 515.1 observed.
**M2** trifluoroacetate salt: Following the general procedure, **6** (58 mg, 204 µmol), AlMe_3_ (2M in toluene, 0.82 mL, 1.63 mmol), and **7b** (56.5 mg, 204 µmol) were reacted to prepare crude **8c** (crude yield 90 mg, 83%). A Schlenk flask was charged with crude **8c** (90 mg) and reacted with BBr_3_ (0.43 g, 0.16 mL, 1.70 mmol) according to the general procedure to obtain **M2** as a yellow solid (57 mg, 54% over two steps). ^1^H NMR (400 MHz, DMSO-*d*_6_, 298 K): 12.11 (br s, 1H), 10.38 (br s, 1H), 9.54 (br s), 9.26 (d, *J* = 1.6 Hz, 1H), 8.77 (s, 1H), 8.64 (m, 2H), 8.03 (d, *J* = 8.1 Hz, 1H), 7.69 (dd, *J* = 8.1, 5.3 Hz, 1H), 7.65 (s, 1H), 7.22 (s, 1H), 6.99 (s, 1H), 6.96 (d, *J* = 8.1 Hz), 3.73 (br s, 2H), 3.42 (br s, 4H), 3.06 (br s, 4H), 2.81 (s, 3H), 2.29 (s, 3H). ^13^C {^1^H} NMR (DMSO-*d*_6_, 126 MHz, 298 K): 166.3, 165.6, 158.6 (q, *J* = 35 Hz), 145.1, 143.6, 142.4, 139.2, 137.9, 136.6, 132.2, 130.6, 129.3, 125.7, 124.2, 120.3, 118.4, 117.1, 116.1 (q, *J* = 295 Hz), 115.8, 113.3, 107.3, 59.4, 51.4, 48.7, 42.1, 17.8. ESI-MS: M + H^+^ = 515.2 expected, 515.1 observed.
**M3** trifluoroacetate salt: Following the general procedure, **6** (130 mg, 461 µmol), AlMe_3_ (2M in toluene, 0.92 mL, 1.84 mmol), and **7d** (75 mg, 507 µmol) were reacted to prepare **M3** (36 mg) in a 15% yield. ^1^H NMR (500 MHz, DMSO-*d*_6_, 298 K): 10.15 (s, 1H), 9.55 (br s, 1H), 9.33 (d, *J* = 1.7 Hz, 1H), 8.80 (s, 1H), 8.79 (d, *J* = 1.8 Hz), 8.69 (dd, *J* = 5.2, 1.2 Hz, 1H), 7.92 (d, *J* = 8.2 Hz, 2H), 7.83 (dd, *J* = 8.2, 5.2 Hz, 1H), 7.70 (s, 1H), 7.34 (d, *J* = 8.1 Hz, 3H), 7.19 (d, *J* = 8.3 Hz, 1H), 2.39 (s, 3H), 2.27 (s, 3H). ^13^C {^1^H} NMR (126 MHz, DMSO d6, 298 K): 165.9, 165.2, 158.3 (q, *J* = 35 Hz), 145.1, 143.6, 143.5, 142.4, 141.5, 139.0, 138.1, 137.9, 137.8, 132.2, 130.4, 128.9, 127.8, 125.7, 123.6, 115.9 (q, *J* = 291 Hz), 115.5, 113.1, 112.9, 107.2, 21.0, 17.6. ESI-MS: M + H^+^ = 401.1 expected, 401.0 observed.

Synthesis of masitinib **M4** derivative:


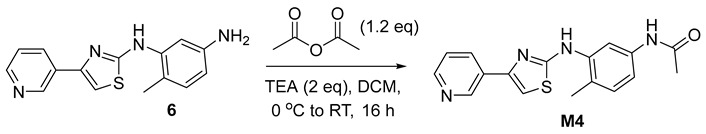


We added to a flame-dried round bottom flask **6** (50 mg, 0.18 mmol), TEA (39 mg, 0.38 mmol), 50 mL of dry DCM, and a stir bar. The reaction was sealed, cooled to 0 °C, and placed under nitrogen, and then, acetic anhydride (22 mg, 0.21 mmol) was added in a single portion. This reaction was warmed to room temperature gradually and stirred for 16 h. The reaction was then extracted with 2 × 50 mL of 0.1 M aqueous NaOH, concentrated under reduced pressure, and redissolved in an ACN:H_2_O + 0.1% TFA solution. The reaction was purified via preparative HPLC using a 5% to 60% ACN in H_2_O + 0.1% TFA gradient elution, and the product-containing fractions were pooled and were freeze-dried to obtain **M4** (36 mg) as a yellow powder in a 61% yield. ^1^H-NMR (400 MHz, DMSO-*d6*): 9.91 (s, 1H), 9.49 (s, 1H), 8.74 (d, 1H), 8.68 (d, 1H), 8.58 (s, 1H), 7.82 (t, 1H), 7.68 (s, 1H), 7.12 (s, 2H), 2.23 (s, 3H), 2.06 (s, 3H). ESI-MS: M + H^+^ = 325.1 expected, 325.2 observed.

Scheme S2 from the Supplementary Materials shows the synthesis of the masitinib **M5** derivative from 2-amino-4-methoxytoluene using a synthetic procedure adapted from Scheme S1 (SI file).

Synthesis of 1-(5-methoxy-2-methylphenyl)thiourea (**9**):

We charged a round bottom flask with ammonium thiocyanate (1.5 g, 20 mmol), acetone (20 mL), and a stir bar under an inert atmosphere. Benzoyl chloride (2.1 mL, 18 mmol) was added dropwise. The reaction mixture was heated to reflux and stirred for 20 min. Then, 5-methoxy-2-methylaniline (2.5 g, 18 mmol) was added portion-wise and stirred for 1 h. Then, the reaction mixture was poured into ice water, and the precipitate was filtered. A round bottom flask was charged with the precipitate, a 2.5 M aqueous NaOH solution (100 mL), and a stir bar under an inert atmosphere. The reaction mixture was heated to reflux and stirred for 1 h. After complete consumption of the start material was confirmed with TLC, the reaction mixture was cooled to 0 °C and filtered. The precipitate was rinsed with water (100 mL) and used in the next step without further purification. The crude product was a grey solid in a 73% yield. ^1^H NMR (400 MHz, DMSO-*d*_6_, 298 K): 9.40 (s, 1H), 7.12 (d, *J* = 8.4 Hz, 1H), 6.83 (d, *J* = 2.7 Hz, 1H), 6.74 (dd, *J* = 8.4, 2.7 Hz, 1H), 3.71 (s, 2H), 2.10 (s, 2H). ^13^C {^1^H} NMR (101 MHz, DMSO-*d*_6_, 298 K): 181.5, 157.5, 137.9, 131.0, 125.9, 112.9, 112.2, 55.1, 16.8.

Synthesis of N-(5-methoxy-2-methylphenyl)-4-(pyridin-3-yl)thiazol-2-amine (**10**):

A round bottom flask was charged with **9** (1.0 g, 5.1 mmol), 2-bromo-1-pyridin-3-ylethan-1-one hydrobromide (1.4 g, 5.1 mmol), potassium bicarbonate (0.72 g, 7.1 mmol) ethanol (71 mL), and a stir bar under an inert atmosphere. The reaction mixture was heated to 75 °C and stirred for 20 h. Then, the reaction mixture was cooled to room temperature; filtered and concentrated under reduced pressure; and redissolved in chloroform (50 mL), which was washed with a saturated aqueous sodium bicarbonate solution (50 mL). The organic phase was dried over MgSO_4_ and purified using silica gel chromatography (33–100% EtOAc in hexanes). The product was a white solid in a 45% yield. ^1^H NMR (400 MHz, CDCl_3_, 298 K): 9.12 (s, 1H), 8.54 (d, *J* = 4.7 Hz, 1H), 8.17–8.03 (m, 1H), 7.39 (s, 1H), 7.31 (dd, *J* = 7.9, 4.7 Hz, 1H), 7.13 (d, *J* = 8.3 Hz, 1H), 6.90 (s, 1H), 6.70–6.56 (m, 1H), 3.83 (s, 3H), 2.27 (s, 3H). 

Synthesis of 4-methyl-3-((4-(pyridin-3-yl)thiazol-2-yl)amino)phenol (**11**):

A round bottom flask was charged with **10** (0.53 g, 1.8 mmol) and dry CH_2_Cl_2_ (17 mL) under an inert atmosphere. The reaction mixture was cooled to 0 °C, and BBr_3_ (1.1 mL, 12 mmol) was added dropwise. The reaction mixture was stirred at room temperature for 16 h. After this time, a 1M aqueous NaOH solution (250 mL) was added to the reaction, which was stirred for 2 h at room temperature. We concentrated the mixture under reduced pressure to remove the CH_2_Cl_2_ and neutralized to pH 7 with a 2M aqueous HCl solution. The mixture was then filtered, and the precipitate was purified using silica gel chromatography (0–10% MeOH in DCM). The product was an off-white solid in a 50% yield. ^1^H NMR (400 MHz, DMSO-*d6*, 298 K): 11.07 (s, 1H), 8.64 (d, 1H), 8.38 (dd, 1H), 7.59 (d, 1H), 7.28 (s, 1H), 7.15 (s, 1H), 7.06 (s, 2H), 7.02 (s, 1H), 6.67 (d, 1H), 6.03 (d, 1H), 2.18 (s, 3H). 

Synthesis of masitinib **M5** derivative: 

To a flame-dried round bottom flask, we added **11** (28 mg, 0.1 mmol), TBTU (32 mg, 0.1 mmol), DBU (50 μL, 0.35 mmol), 5 mL of dry DMF, and a stir bar. The vessel was sealed and stirred for 10 min; then, **10** (25 mg, 0.1 mmol) dissolved in 2 mL of dry DMF was added in a single portion. The reaction was allowed to proceed for 4 h, at which point LC-MS confirmed successful trans-esterification. The crude reaction mixture was concentrated and purified using preparative HPLC (5–60% gradient elution of ACN in H_2_O + 0.1% TFA). The product fractions were pooled and freeze-dried to obtain **M5** (21 mg) as a yellow powder in a 42% yield. ^1^H-NMR (400 MHz, DMSO-*d*_6_, 293 K): 9.64 (s, 1H) 9.12 (s, 1H), 8.57 (dd, 1H), 8.43 (dt, 1H), 8.15 (m, 3H), 7.64 (s, 1H), 7.59 (m, 3H), 7.31 (d, 1H), 6.91 (dd, 1H), 3.79 (s, 2H), 3.41 (br s*, 4H), 3.04 (br s*, 4H), 2.33 (s, 3H). ESI-MS: M + H^+^ = 500.2 expected, 500.3 observed. (* = broad singlets were observed due to poor rotation of the piperazine bond and associated protons at 293 K).

## Figures and Tables

**Table 1:** Ligand–protein H-bond interactions. In all cases, we report the fraction of time that a given interaction occurs based on geometrical criteria: heavy atom distance below 3.5 Å, and donor–hydrogen–acceptor angle higher than 140°. In both cases, M(wt)^1^ refers to masitinib with a net charge equal to +1 (with its *N*-methylpiperazine group protonated) while M(wt)^0^ refers to masitinib in its uncharged state.

Protein	Molecule	Acceptor	Donor	Fraction
M^pro^ (SARS-CoV-2)	M(wt)^1^	His164 (O)	LIG (N1)	0.82
		LIG (N3)	His163 (NE2)	0.56
		Thr24	LIG	0.07
	M(wt)^0^	His164 (O)	LIG (N1)	0.94
		LIG (N3)	His163 (NE2)	0.53
		LIG	Thr24	0.12
M^pro^ (OC43)	M(wt)^1^	Gln164 (O)	LIG (N1)	0.75
		LIG (N3)	His163 (NE2)	0.44
		Asn24	LIG	0.35
	M(wt)^0^	Gln164 (O)	LIG (N1)	0.56
		LIG (N3)	His163 (NE2)	0.20
		LIG	Glu166	0.12

**Table 2:** Absolute binding free energy calculations: M^pro^ from SARS-CoV-2 as molecular target. The average free energy values and their standard deviations were estimated from four independent replicas. LIE-D: Linear interaction energy estimated as proposed by Miranda et al. [18]. TI: Thermodynamic integration.

Ligand	LIE-D (kcal/mol)	TI (kcal/mol)	S.D
M_wt_	−12.52	−8.80	0.80
**M1**	−12.22	−9.28	1.28
**M2**	−13.41	−10.06	1.47
**M3**	−11.42	−8.35	1.13
**M4**	−10.95	−6.66	1.78
**M5**	−11.57		

**Table 3:** Masitinib derivatives exhibit reduced cytostatic activity in A549 cells. The doubling time of A549 cells in the presence of DMSO or masitinib and its derivatives was calculated after fitting an exponential curve through data points generated from Figure 2A. ND = not determined.

Doubling Time (h)		Dose			
		10 µM	3 µM	1 µM	0.3 µM
COMPOUNDS	DMSO	23.13	ND	ND	ND
	M_WT_	347.8	74.57	24.94	22.72
	M1	52.78	23.72	21.89	21.75
	M2	33.63	21.57	21.44	21.52
	M3	30.76	22.49	21.49	22.13
	M4	37.36	22.28	21.47	22.01
	M5	25.19	21.83	21.95	22.37

## Data Availability

Not applicable.

## Supplementary Materials

The following supporting information can be downloaded at https://www.mdpi.com/article/10.3390/molecules28186643/s1, Figure S1: Structural comparison between M^pro^ from SARS-CoV-2 and HCoV-OC43; Figure S2: Estimation of the root mean square fluctuations (RMSFs) for masitinib bound to M^pro^, Figure S3: Root mean square deviation of atomic positions, Figure S4: Structural properties of the key hydrogen bond interactions displayed by masitinib with His163 and His164. Table S1: Ligand–protein H-bond interactions, Figure S5: Ligand–protein 2D contact map. Figure S6: Root mean square deviation of atomic positions. Scheme S1: Synthesis of masitinib, **M1**, **M2**, and **M3.** Scheme S2: Synthesis of masitinib **M5**.

## Sample Availability

Samples of the compounds are available from the authors upon request.
