# Supplementary material for: Development of Masitinib Derivatives with Enhanced Mpro Ligand Efficiency and Reduced Cytotoxicity
Source: Molecules. 2023 Sep 15;28(18):6643. doi: 10.3390/molecules28186643 (PMC10536273; doi:10.3390/molecules28186643)
Supplement: Supplementary file 1 [file molecules-28-06643-s001.zip › molecules-2564820-supplementary.pdf]

Supporting information

Development of Masitinib Derivatives with Enhanced M^{pro} Ligand Efficiency and Reduced Cytotoxicity

Cintia A. Menendez [1], Adil Mohamed [1], Gustavo R. Perez-Lemus [1], Adam M. Weiss [2], Benjamin W. Rawe [1],
Guancen Liu [2], Alex E. Crolais [2], Emma Kenna [1], Fabian Byléhn [1], Walter Alvarado [1], Dan Mendels [1],
Stuart J. Rowan [1,2], Savaş Tay [1] and Juan J. de Pablo [1,3,*]

[1] Pritzker School of Molecular Engineering, University of Chicago, 5640 South Ellis Avenue, Chicago, IL 60637, USA; menendezc@uchicago.edu (C.A.M.); adilm@uchicago.edu (A.M.); grp@uchicago.edu (G.R.P.-L.); brawe@uchicago.edu (B.W.R.); ekenna@uchicago.edu (E.K.); bylehn@uchicago.edu (F.B.); walt@uchicago.edu (W.A.); danmendels@uchicago.edu (D.M.); stuartrowan@uchicago.edu (S.J.R.); amannie@uchicago.edu (S.T.)
[2] Department of Chemistry, University of Chicago, 5735 South Ellis Avenue, Chicago, IL 60637, USA; adamweiss@uchicago.edu (A.M.W.); guancen123@uchicago.edu (G.L.); acrolais@uchicago.edu (A.E.C.)
[3] Argonne National Laboratory, 9700 South Cass Avenue, Lemont, IL 60439, USA
* Correspondence: depablo@uchicago.edu

1-Molecular dynamics simulation:

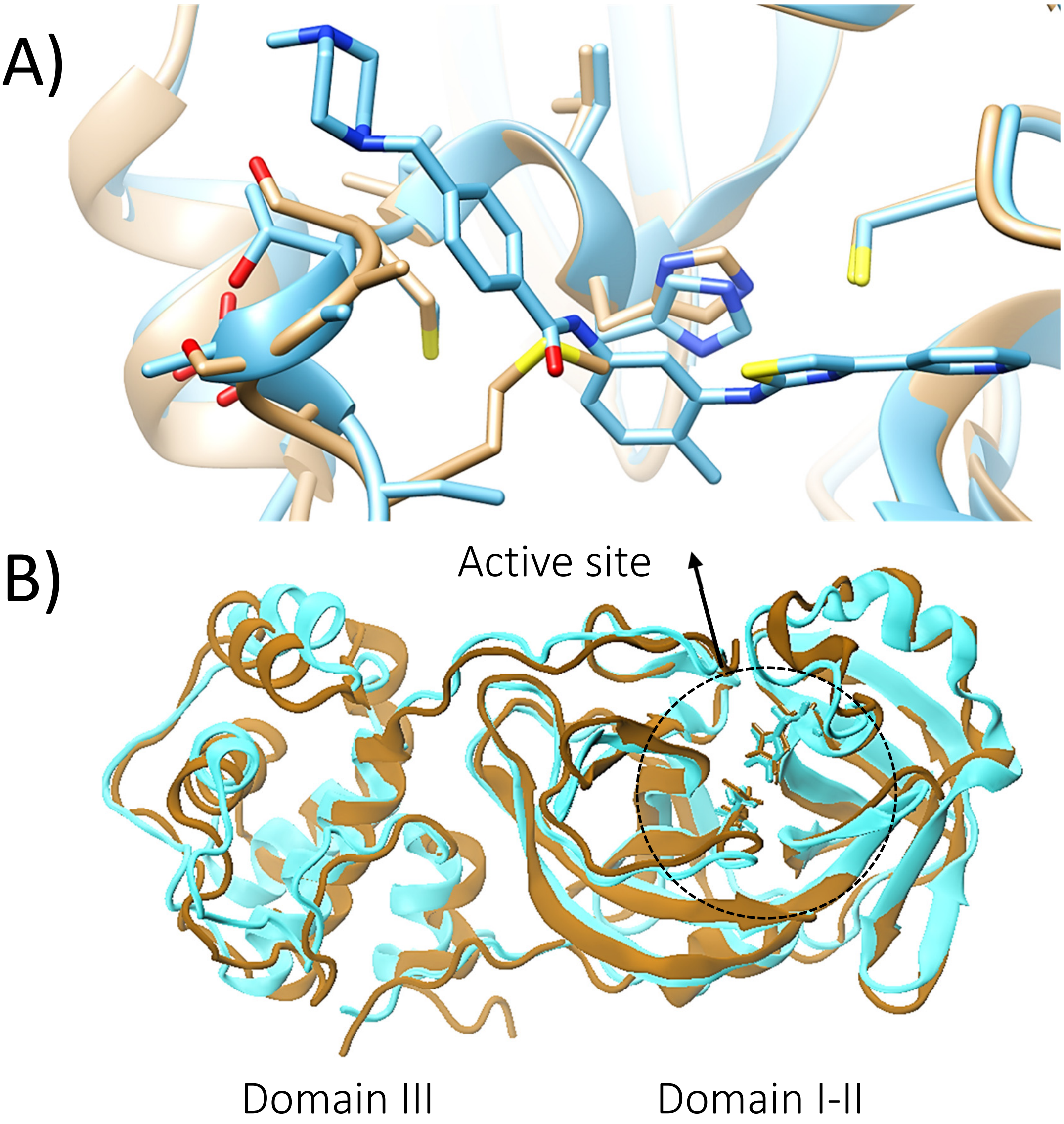

Figure S1. A structural comparison between M^{pro} from SARS-CoV-2 and HCoV-OC43: A) M^{pro} active site from HCoV-OC43 (cyan) and SARS-CoV-2 (light brown). The catalytic residues, His41 and Cys145 are shown as sticks. Masitinib is shown with sticks where Nitrogen atoms are colored in blue, Carbone in cyan, Oxygen in red and Sulfur in yellow. A close contact between Met49 and Masitinib is observed from the direct alignment. B) Ribbons representation for M^{pro} monomer alignment: HCoV-OC43 (cyan) and SARS-CoV-2 (light brown). The catalytic residues are depicted as sticks in both cases.

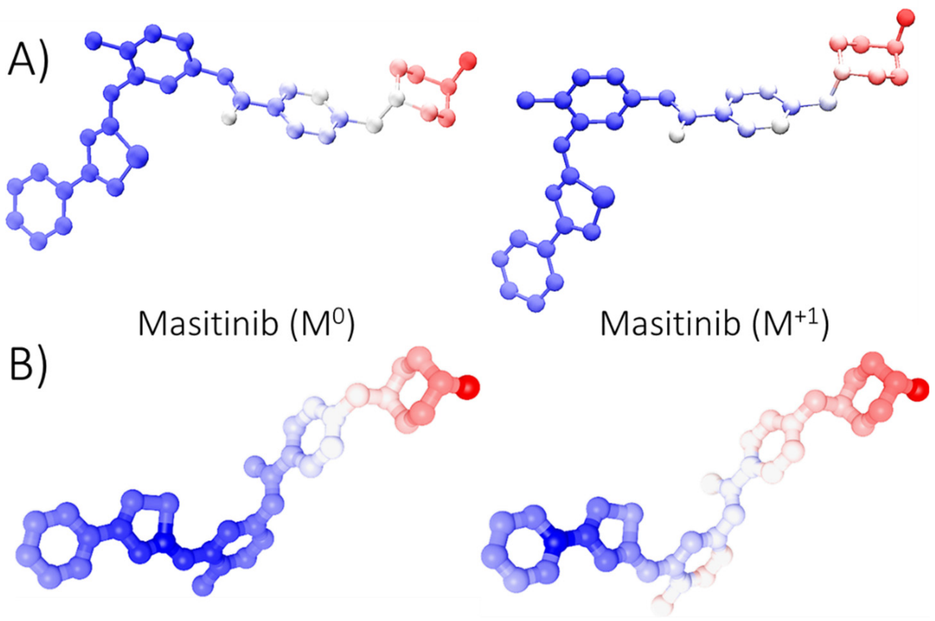

Figure S2. ***Estimation of the Root Mean Squared Fluctuations (RMSF) for Masitinib bound to M^{pro}*:** A) M^{pro} (SARS-CoV-2)-masitinib complex. B) M^{pro} (OC43)-masitinib complex. In both cases, the chemical structure of the ligand is represented with CPK (VMD representation method) and each ligand heavy atom is colored by its RMSF individual value, where the lowest values are shown in blue (low thermal fluctuations) and the highest values are depicted in red (high thermal fluctuations). In both systems, uncharged masitinib is on the left while the protonated ligand (charge= +1) is on the right.

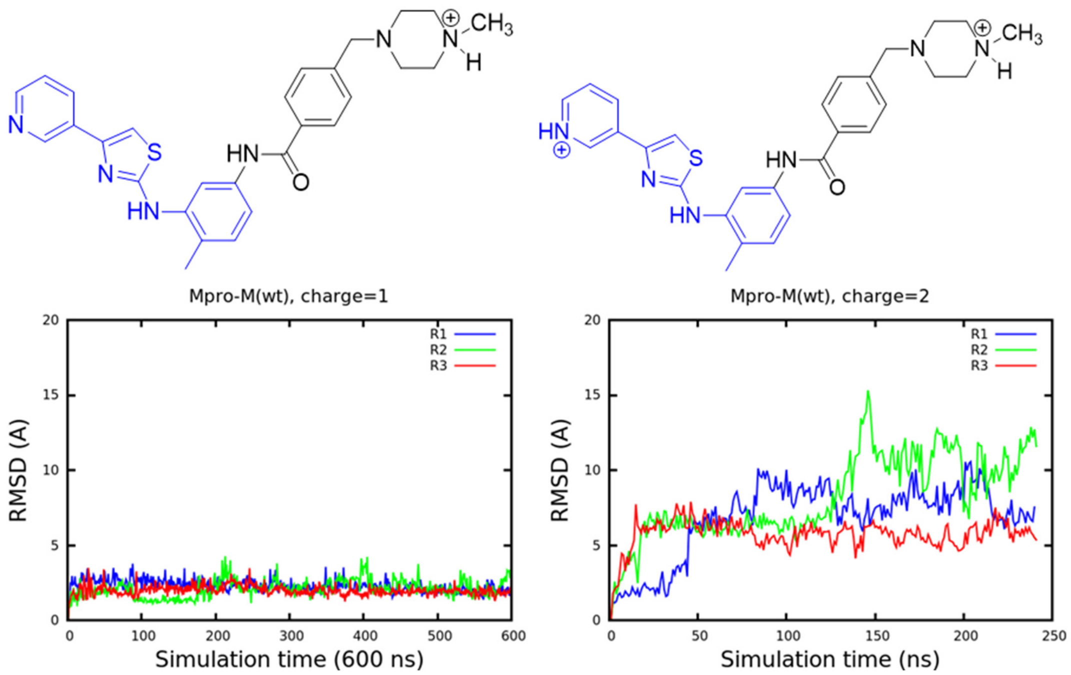

Figure S3. Root Mean Square Deviation of atomic positions: RMSD for the ligand heavy atoms as function of the simulation time in three independent replicas for Masitinib with net charge equal to +1 (left) and +2 (right).

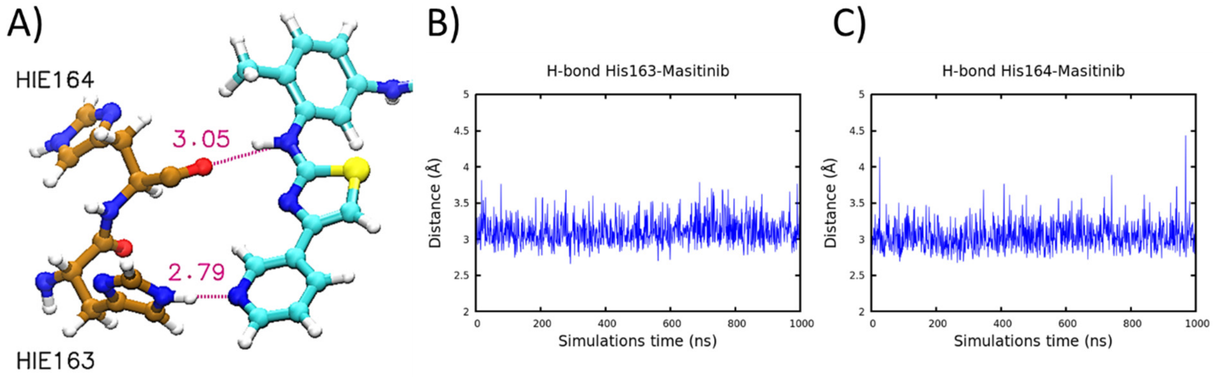

Figure S4. Structural properties of the key hydrogen bond interactions display by masitinib with His163 and His164: A) Representative configuration of the Masitinib-M^{pro} complex where are highlight the main anchoring points of Masitinib. Hydrogen bonds are represented as dotted magenta lines and the heavy distance (Å) is also shown in the same color. The key residues His^{163} and His^{164} are shown in brown whereas Masitinib (a fragment for simplicity) is depicted in cyan. The color code for heteroatoms is Nitrogen (blue), Oxygen (red) and Sulfur (yellow). B) and C) Hydrogen bonds structural properties as function of simulation time: in both cases are represented the distance between the heavy atoms displaying the interaction. This is: His^{163}(**NE2**)-M^{wt}(**N3**) in the case of figure B and His^{164}(**O**)-M^{wt}(**N1**) in figure C.

Protein	*Molecule*	*Acceptor*	*Donor*	*Frac*
M^{pro} (SARS-CoV-2)	*M1*	HIS 164(O)	LIG (N1)	0.941
		LIG (N3)	HIS 163 (NE2)	0.692
		THR 25(OG1)	LIG (NBD)	0.564
		SER 46 (OG)	LIG (NBD)	0.169
	M2	HIS 164 (O)	LIG (N1)	0.920
		LIG (N3)	HIS 163 (NE2)	0.729
		THR 25 (OG1)	LIG (NBD)	0.665
		CYS 44 (O)	LIG (O1)	0.619
	M3	HIS 164 (O)	LIG (N1)	0.792
		LIG (N3)	HIS 163 (NE2)	0.629
		GLN 189 (OE1)	LIG (NAT)	0.166
	M4	HIS 164 (O)	LIG (NAL)	0.950
		LIG (NAD)	HIS 163 (NE2)	0.521
		MET 49 (O)	LIG (NAT)	0.186
	M5	GLU 166 (O)	LIG (NAL)	0.406
		LIG (NAD)	HIE 163 (NE2)	0.289
		HIE 164 (O)	LIG (NBG)	0.162

Table S1. *Ligand-Protein H-bond interactions:* in all cases, it is reported the fraction of time that a given interaction is formed based on geometrical criteria: heavy atoms distance lower than 3.5 Å and the donor-hydrogen-acceptor angle higher that 140°. The reported quantities correspond to the average from three independent replicas of at least 600 ns each of them.

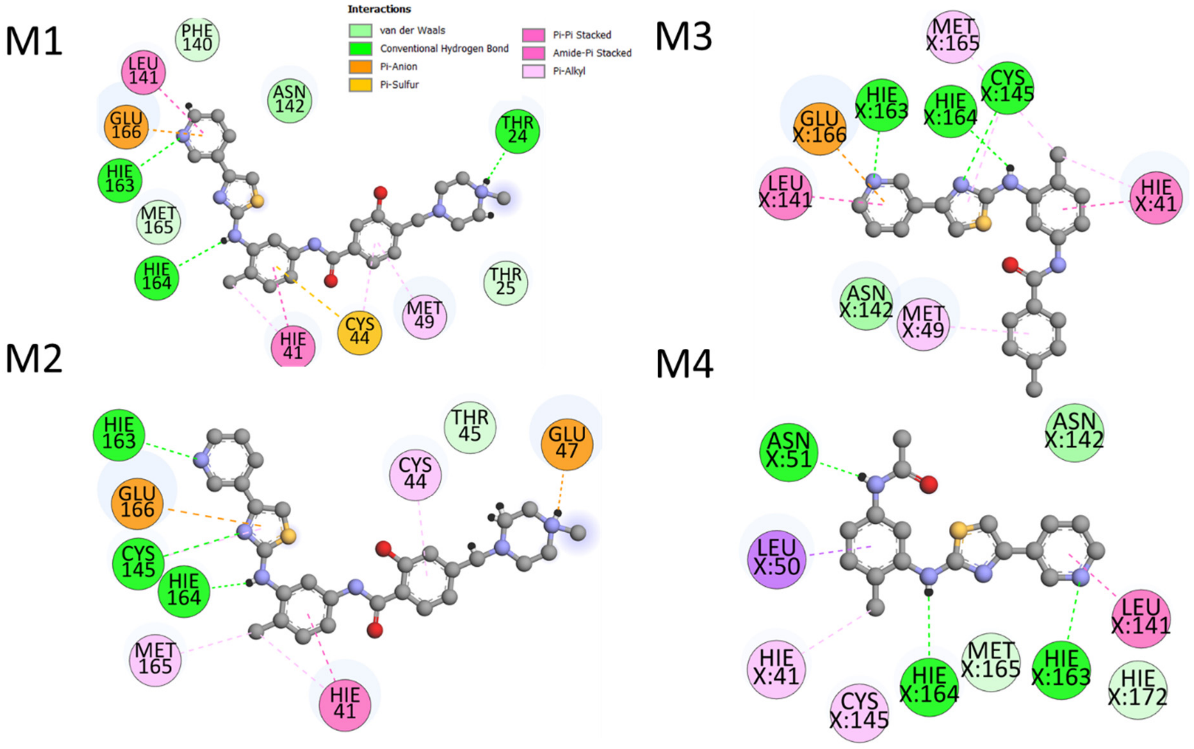

Figure S5. ***Ligand-protein 2D contact map***: the interactions that **M1-M4** (shown as chemical structures) make with M^{pro} residues. The colors of the interactions correspond to the color shown in the legend. The interaction maps were prepared with BIOVIA Discovery Studio Visualizer.

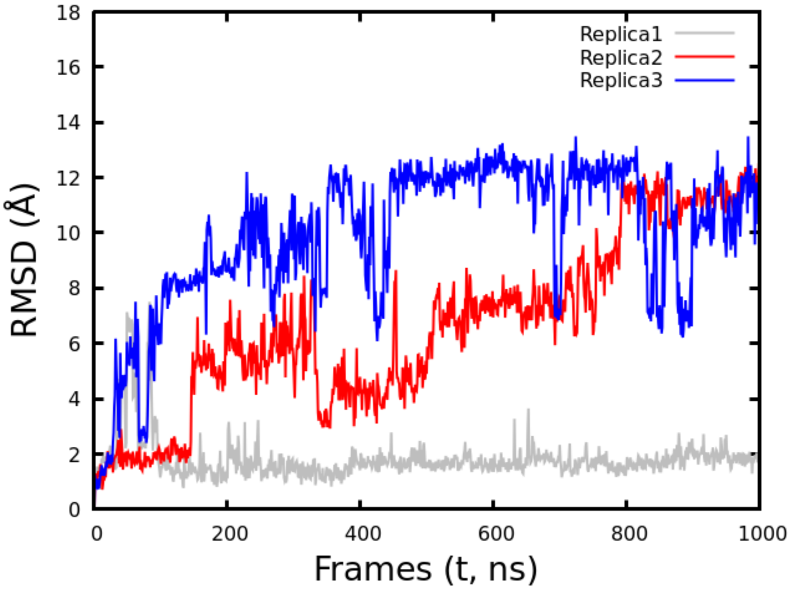

Figure S6. Root Mean Square Deviation of atomic positions: RMSD for the ligand heavy atoms as function of the simulation time in three independent replicas for Masitinib derivative **M5.**

2- Organic Chemistry:

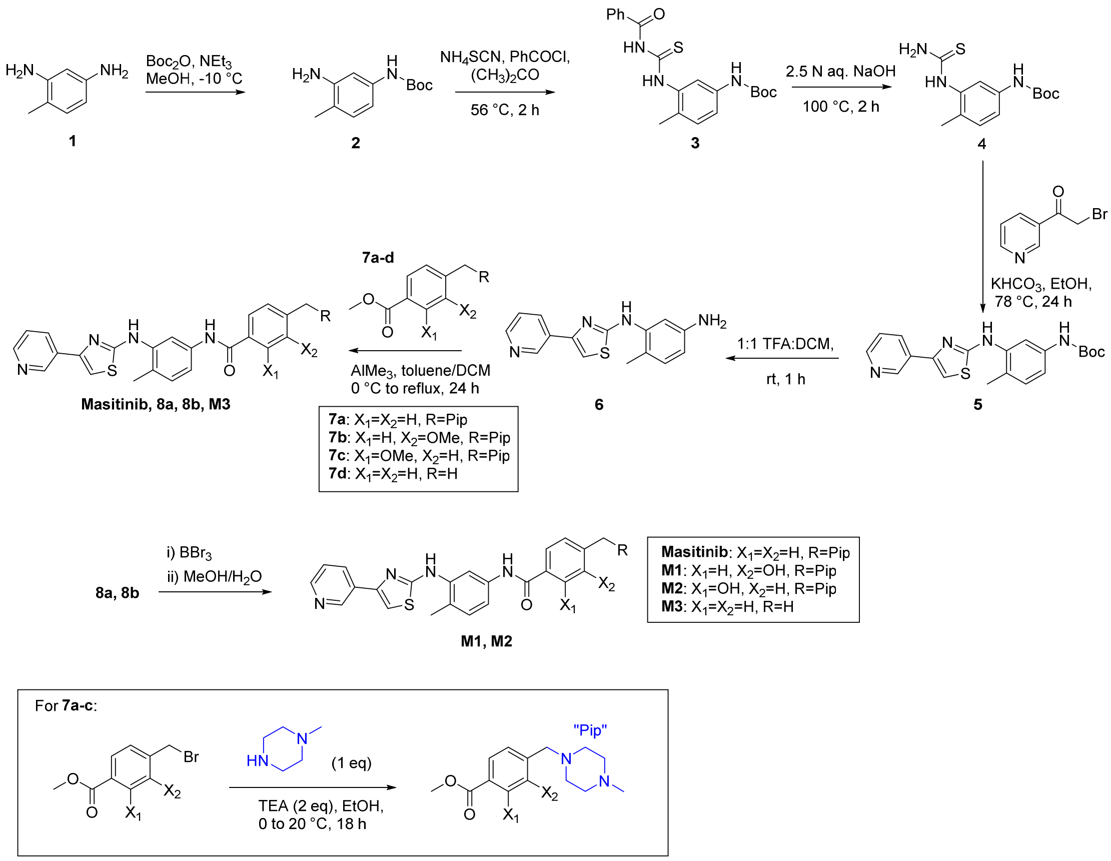

Scheme S1. Scheme for the synthesis of Masitinib, **M1**, **M2**, and **M3** adapted from [S1]. The key chemical transformation involves amidation of **6** with the esters **7a-d** using $AlMe_3$ followed by deprotection with BBr_3 to form compounds with modifications at the X_1, X_2, and R sites.

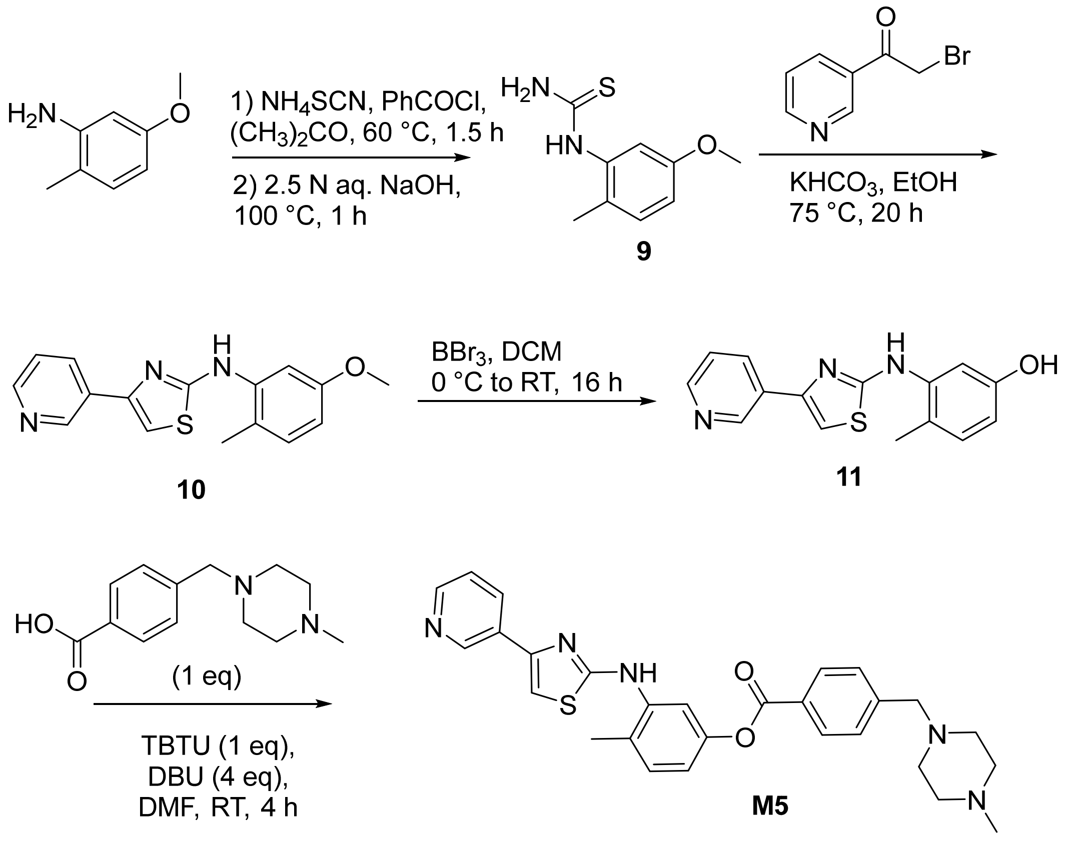

Scheme S2. Scheme for the synthesis of Masitinib **M5** derivative from 2-amino-4-methoxytoluene using a synthetic procedure adapted from **Scheme S1**.

References

[S1] Ciufolini, M.; Wermuth, C.; Gielthen, B.; Moussy, A. 2-(3-aminoaryl)amino-4-aryl-thiazoles and their use as c-kit inhibitors. W.O. Patent 2004/014903 A1, February 19, 2004.
